# Age Differences in Psychological Impact of Cancer and Access to Psychotherapy in Nigeria: A National Study.

**DOI:** 10.1093/geroni/igaf122.759

**Published:** 2025-12-31

**Authors:** Runcie Chidebe, Sampson C Ipiankama, Maria Chidi C Onyedibe, Darlingtina Esiaka, JohnBosco Chika Chukwuorji

**Affiliations:** Miami University, Oxford, Ohio, United States; Project PINK BLUE - Health & Psychological Trust Centre, Abuja, Federal Capital Territory, Nigeria; University of Nigeria Nsukka Department of Psychology, Nsukka, Enugu, Nigeria; University of Kentucky - College of Medicine, Lexington, Kentucky, United States; Michigan State University, East Lansing, Michigan, United States

## Abstract

The cumulative risk of developing and dying from cancer before the age of 75 years is 12% and 8.0% in Nigeria. While certain cancer types (e.g., prostate, liver, cervical, and breast) more commonly affect people ages 30+, the age of cancer diagnosis is low in Nigeria compared to many countries. Yet, the psychological impact of cancer diagnosis among younger and older adults is largely unknown. We aimed to estimate the age difference in the psychological impact of a cancer diagnosis and access to psychotherapy and psycho-oncologists among cancer patients receiving treatment (i.e., chemotherapy, radiotherapy, and immunotherapy) across all the cancer hospitals in Nigeria. Cancer patients (N = 1,374, Mage=52.15) completed the national access to psychotherapy and psycho-oncology questionnaire. We analyzed the data using multiple linear regression and Fisher’s discriminant functions. The result showed that older adults (M = 53.81, SD = 14.58) were more likely than younger adults (M = 51.76, SD = 12.58) to be affected psychologically when they received the news of their cancer diagnosis, F(1, 1317) = 4.31, p=.038. However, there was no age difference in their receiving psychological services after they learned of their cancer, access to psychological support during treatment, need to see a psychologist during treatment, utilization of psychological services during treatment and knowledge of where they could receive psychological or counseling services. These findings highlight the need for targeted psychological interventions to support older adults in coping with the emotional impact of a cancer diagnosis in Nigeria.

